# Clinical and Echocardiographic Predictors of New-Onset Atrial Fibrillation in Patients with Non-AF Arrhythmias: An Exploratory Analysis of NT-proBNP

**DOI:** 10.3390/biomedicines14061252

**Published:** 2026-05-30

**Authors:** Vinh Thanh Tran, Linh Ha Khanh Duong, Sang Doan, Nien Vinh Lam, Dung Ngoc Kieu, Thuc Tri Nguyen

**Affiliations:** 1 Department of Pathology and Gastro-Hepato Integrated Research Team (GHIRT-002.TCM2025), University of Medicine and Pharmacy at Ho Chi Minh City, Ho Chi Minh City 700000, Vietnam; vinhthanhtran2002@gmail.com (V.T.T.); nien@ump.edu.vn (N.V.L.); 2 Department of Biochemistry, Cho Ray Hospital, Ho Chi Minh City 700000, Vietnam; 3 Department of Ophthalmic Imaging and Functional Exploration, Eye Hospital of Ho Chi Minh City, Ho Chi Minh City 700000, Vietnam; bssangoph@gmail.com; 4 Department of Arrhythmia Treatment, Cho Ray Hospital, Ho Chi Minh City 700000, Vietnam; bacsidung@gmail.com; 5 Ministry of Health, Hanoi 100000, Vietnam; ntthucbvcr@gmail.com

**Keywords:** atrial fibrillation, arrhythmia, NT-proBNP, prediction

## Abstract

**Background/Objectives:** Atrial fibrillation (AF) is a prevalent arrhythmia associated with severe clinical complications. This exploratory study aimed to evaluate the prognostic value of clinical characteristics, echocardiographic parameters, and N-terminal pro-B-type natriuretic peptide (NT-proBNP) in predicting new-onset AF among patients previously diagnosed with non-AF arrhythmias. **Methods:** A prospective cohort study was conducted involving 232 patients who were followed for a median period of 12 months. Data collection included baseline NT-proBNP levels, demographic characteristics, medical history, presenting clinical symptoms, and various paraclinical indices, including echocardiographic measurements. **Results:** The most frequent presenting symptoms in the study population were syncope (59.9%) and dizziness (55.6%). Statistical analysis indicated that initial NT-proBNP levels were not a significant predictor for the development of new-onset AF (HR = 0.9995; *p* = 0.717). Conversely, left atrial (LA) size, a history of diabetes mellitus, and a history of stroke were identified as preliminary risk factors requiring confirmation. Specifically, a history of stroke was associated with a nearly 5-fold increase in AF risk (HR = 4.65), while diabetes mellitus increased the risk nearly 4-fold (HR = 3.84). Furthermore, each one-millimeter increase in LA size was associated with a 21% increase in the risk of developing AF (HR = 1.21). **Conclusions:** The findings suggest that NT-proBNP is not an effective prognostic marker for new-onset AF in patients with non-AF arrhythmias. Instead, left atrial enlargement and clinical comorbidities, such as diabetes and a history of stroke, emerged as suggestive, hypothesis-generating predictors for clinical screening and risk management in this patient population.

## 1. Introduction

Atrial fibrillation (AF) is one of the most common clinical arrhythmias, characterized by chaotic electrical activity and irregular contraction of the atria [1,2]. This condition can lead to several serious complications such as stroke, heart failure, and an increased risk of mortality [1,3]. In particular, the early and accurate prognosis of AF risk in patients with other arrhythmias (excluding AF) presents a significant challenge. Timely identification of risk factors and biomarkers could help to personalize treatment and prevention strategies more effectively.

In recent years, considerable research efforts have been dedicated to identifying biomarkers and clinical factors capable of predicting the onset of AF [4,5,6,7,8]. Among these, natriuretic peptides, especially N-terminal pro-B-type natriuretic peptide (NT-proBNP), have garnered significant attention. NT-proBNP is a biomarker released from the ventricles in response to myocardial wall stress and volume overload and is widely used for the diagnosis and prognosis of heart failure [9,10].

Previous studies have shown that elevated NT-proBNP levels are associated with a higher risk of developing AF in the general population, as well as in patients with heart failure or structural heart disease [4,11,12,13]. Proposed mechanisms include the effects of increased ventricular filling pressure, atrial remodeling, inflammation, and myocardial fibrosis, all of which contribute to the pathophysiology of AF. Several studies have demonstrated the independent role of NT-proBNP in predicting new-onset or recurrent AF after interventional procedures [4,11,12,13]. However, data on the role of NT-proBNP in predicting AF in the specific population of patients already diagnosed with other arrhythmias (non-AF) remains limited and has not been fully elucidated.

This exploratory study was conducted to evaluate the predictive role of baseline clinical profiles, left atrial structure, and plasma NT-proBNP levels for the onset of AF in patients with non-AF arrhythmias. By repositioning the focus on both established clinical risk factors and biomarkers, this study aims to provide multidimensional scientific evidence to optimize screening, monitoring, and AF risk management strategies in this specific population, thereby helping to improve patient clinical outcomes.

## 2. Materials and Methods

### 2.1. Study Design and Population

This study was designed as a prospective cohort study conducted at the Department of Arrhythmia Treatment, Cho Ray Hospital, from December 2024 to June 2026. We followed a group of patients with non-atrial fibrillation (non-AF) arrhythmias to evaluate the incidence of new-onset AF and associated factors over time, with a specific focus on the relationship with baseline NT-proBNP levels.

The study population consisted of patients diagnosed with cardiac arrhythmia and treated at the department. Specific inclusion criteria were:

Patients aged 18 years or older.

Diagnosed with a non-AF arrhythmia upon hospital admission, based on 12-lead electrocardiogram (ECG), Holter ECG, or other cardiac monitoring methods.

Had plasma NT-proBNP levels measured at the time of admission.

Provided informed consent to participate in the study.

Patients were excluded if they had conditions that could significantly influence the study results or complicate follow-up, including:

A prior history of AF or an AF diagnosis at the time of admission.

Complex or acute cardiac conditions such as severe obstructive hypertrophic cardiomyopathy, severe aortic valve stenosis, or acute myocardial infarction within 7 days.

End-stage renal disease (eGFR < 30 mL/min/1.73 m^2^) or undergoing hemodialysis.

Refused to participate or had other severe medical conditions (e.g., end-stage cancer, severe liver failure) that precluded complete follow-up.

### 2.2. Sample Size

The sample size was calculated based on the primary objective of predicting the incidence of AF, using survival analysis. The formula for the minimum sample size in a survival study, based on the hazard ratio (HR), was applied. Based on a study by Staerk et al. (2020) [13], we used an HR for NT-proBNP of 1.73. The cumulative incidence of AF in patients with arrhythmia was referenced from a study by Lindberg et al. [14], which was 36.8%.

Substituting these values into the formula, we calculated an initial sample size of 160 patients. Accounting for an estimated 30% patient dropout rate during follow-up, we recruited a minimum of 207 patients.

However, the actual observed incidence of new-onset AF in our cohort during the 12-month follow-up was only 6.9% (16 events out of 232 patients). This substantial discrepancy between the hypothesized and observed event rates indicates that the study is significantly underpowered to detect small effect sizes for continuous biomarkers like NT-proBNP in a multivariable context.

### 2.3. Data Collection and Measurement

At the time of hospital admission, baseline clinical information was collected from all patients, including:

Demographics and medical history: Age, sex, history of cardiovascular diseases, and comorbidities (hypertension, diabetes mellitus, heart failure, chronic kidney disease, thyroid disease).

Clinical examination and basic measurements: Blood pressure, heart rate, body weight, height, and Body Mass Index (BMI).

Laboratory and imaging tests: 12-lead ECG to assess rhythm and conduction parameters; echocardiogram to evaluate cardiac function and structure; and basic hematology and biochemistry tests, including renal function (Creatinine, eGFR) and electrolytes.

LA anteroposterior (AP) diameter, measured from the parasternal long-axis view using M-mode or 2D echocardiography.

NT-proBNP Measurement: A venous blood sample was drawn at the time of admission, before any intervention that could affect NT-proBNP levels. NT-proBNP concentration was measured using a quantitative immunoassay on the ADVIA Centaur XPT system from Siemens (Erlangen, Germany). Values were originally reported in pmol/L in accordance with our institution’s standardized central laboratory reporting protocol. According to the manufacturer, the recommended clinical decision thresholds for this specific assay are 14.75 pmol/L (125 pg/mL) for patients younger than 75 years, and 53.1 pmol/L (450 pg/mL) for patients 75 years and older. All assays adhered to the stringent quality control protocols of the accredited laboratory.

Primary Outcome: The primary outcome was the occurrence of new-onset AF during the follow-up period. AF was defined as having irregular atrial electrical activity and a loss of P waves, lasting at least 30 s, and documented on a 12-lead ECG or Holter ECG [15]. Patients were followed up periodically after hospital discharge via a standardized protocol applied uniformly across all study participants. Routine clinical evaluations and standard 12-lead ECGs were scheduled at 1 week, 1 month, and 3 months post-discharge, and every 6 months thereafter, unless special clinical circumstances required more frequent monitoring. In contrast to the fixed clinical visits, Holter ECG monitoring was not strictly protocolized at predetermined time points; instead, it was suspicion-driven, being indicated and performed whenever patients presented with suggestive symptoms (e.g., unexplained palpitations, worsening dizziness, or syncope) or when the treating physician clinically suspected an arrhythmia recurrence. To ensure data reliability, all ECG and Holter ECG recordings were independently interpreted by two experienced cardiologists. Echocardiograms were performed by certified technicians and physicians following international guidelines.

### 2.4. Statistical Analysis

Statistical analyses were performed using R software (version 4.5.3; R Foundation for Statistical Computing, Vienna, Austria). Continuous variables were expressed as mean ± standard deviation (SD) for normally distributed data or median (interquartile range, IQR) for skewed distributions, with normality assessed via the Shapiro–Wilk test. Categorical variables were presented as absolute frequencies and percentages.

Between-group comparisons for continuous data were conducted using Student’s t-test or the Mann–Whitney U test, as appropriate. Categorical data were compared using the Pearson’s chi-squared test or Fisher’s exact test for small cell counts. Regarding missing data, all baseline clinical, laboratory, and echocardiographic covariates utilized in the analysis were 100% complete at admission; thus, a complete-case approach was maintained throughout, and no data imputation was required. The cumulative incidence of atrial fibrillation (AF) over the follow-up period was estimated using the Kaplan–Meier method, with differences evaluated via the log-rank test.

To identify independent predictors of AF, a parsimonious prespecified multivariable Cox proportional hazards regression model was constructed. To minimize the risk of overfitting and coefficient instability due to the low absolute number of outcome events (*n* = 16), the number of predictors was strictly constrained to four highly relevant variables selected based on clinical significance and primary hypotheses: baseline NT-proBNP levels, LA size, history of diabetes mellitus, and a history of stroke.

The proportional hazards assumption for the Cox models was formally evaluated and verified using the Schoenfeld residuals test, confirming that the assumption was met for all individual covariates and the global model (*p* > 0.05).

All statistical tests were two-sided, and a value of *p* < 0.05 was considered statistically significant.

### 2.5. Ethical Considerations

The study was approved by the Institutional Ethics Committee of the University of Medicine and Pharmacy, Ho Chi Minh City. All participating patients provided written informed consent after receiving a detailed explanation of the study.

## 3. Results

### 3.1. Baseline Characteristics of the Study Population

The study initially collected data from 285 patients. After excluding 41 patients with a pre-existing history of atrial fibrillation (AF) and 12 patients with severe renal impairment (eGFR < 30 mL/min/m^2^), a total of 232 patients without baseline AF were eligible for participation. The median follow-up duration was 12 months, with an interquartile range (IQR) of 6.75 to 13 months.

The study group comprised 124 males (53.4%) and 108 females (46.6%). The mean age for the entire cohort was 63.7 ± 14.0 years, with a median of 67. Notably, females had a significantly higher mean age than males (67.5 ± 14.4 years vs. 60.4 ± 16.7 years). Other anthropometric indices reflected the biological differences between the sexes: males were taller (1.63 ± 0.05 m) and heavier (61.4 ± 10.2 kg) than females (1.55 ± 0.04 m and 53.3 ± 9.19 kg, respectively). The mean BMI for the cohort was 22.5 ± 3.34 kg/m^2^, which falls within the normal range. The mean blood pressure was also stable, with a systolic pressure of 129 ± 19.6 mmHg and a diastolic pressure of 75.1 ± 10.2 mmHg. However, the mean blood pressure tended to be slightly higher in females, which could be related to their higher mean age (Table 1).

**Figure 1:**
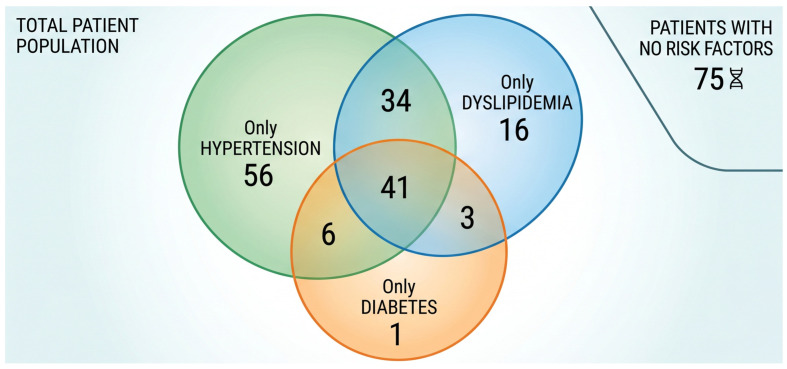
Venn Diagram of Common Cardiovascular Risk Factors Among Patients. This diagram illustrates the overlap and distribution of the three most prevalent cardiovascular comorbidities—hypertension (59.0%), dyslipidemia (40.5%), and diabetes mellitus (22.0%)—among the 232 non-atrial fibrillation arrhythmia patients in the study cohort.

Regarding medical history, the most prevalent cardiovascular risk factors were hypertension (59.0%), dyslipidemia (40.5%), and diabetes mellitus (22.0%). Heart failure (18.0%), coronary artery disease (13.4%), and chronic kidney disease (10.3%) were also common. Of the 232 patients, 31.5% presented with a single cardiovascular risk factor, 18.5% had a co-occurrence of two risk factors, and 17.7% exhibited three or more concomitant risk factors, demonstrating a high multi-comorbidity burden in this arrhythmia cohort. The presenting symptoms for this patient cohort were highly varied, with syncope (59.9%) and dizziness (55.6%) being the most common, each affecting more than half of the patients (Figure 1, Table 2).

Given the heterogeneous nature of the “non-AF arrhythmia” inclusion criteria, we further stratified the cohort based on their specific primary arrhythmia diagnosis or primary indication for electrophysiological intervention at admission. The distribution of these underlying electrophysiological disorders is detailed in Table 3. The most common baseline arrhythmias were atrioventricular (AV) blocks and conduction diseases (37.9%), followed closely by sick sinus syndrome and sinus node dysfunctions (29.7%). This categorization reflects a diverse array of arrhythmic and structural substrates that may differentially influence baseline biomarker levels and future AF risk.

### 3.2. Laboratory and Paraclinical Findings

**Figure 2:**
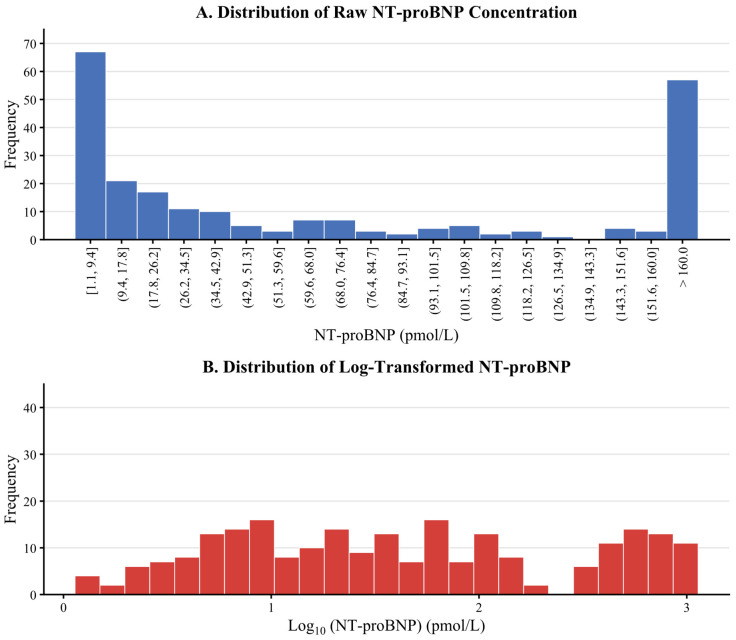
Distribution of NT-proBNP concentration (pmol/L) in 232 non-atrial fibrillation arrhythmia patients participating in the study. (**A**) Distribution of raw NT-proBNP concentration. (**B**) Distribution of log-transformed NT-proBNP. The histograms demonstrate the highly skewed nature of the biomarker’s baseline levels, which are characterized by a massive concentration of patients near baseline values alongside a small, highly elevated cluster that statistically failed to achieve a normal distribution even after logarithmic transformation.

The distribution of NT-proBNP levels in the study cohort was heterogeneous, with most patients having low concentrations while a smaller subgroup had very high levels. The median NT-proBNP value was 34.5 pmol/L [292.2 pg/mL] (IQR: 7.2–157 pmol/L [61.0–1329.8 pg/mL]) (Figure 2). Given the markedly skewed and heterogeneous distribution of the biomarker, secondary mathematical transformations—including natural logarithmic and base-10 logarithmic transformations—were exploratory executed. However, due to the extreme polarization of the data, characterized by a massive concentration of patients near baseline values alongside a small, highly elevated cluster, the log-transformed values still statistically failed to achieve a normal distribution. Consequently, the raw continuous values were retained in the primary model to preserve direct clinical interpretability, while the non-normal nature was clinically addressed via the quartile-based approach.

Most other laboratory values were within normal limits. The median Creatinine was 0.87 mg/dL and the median eGFR was 83.9 mL/min/1.73 m^2^, reflecting an acceptably preserved renal function. Blood lipid levels (Cholesterol, LDL, HDL, Triglyceride) were also within the average range. On echocardiography, the median left atrial (LA) size was 31 mm and the median ejection fraction (EF) was 65%, indicating that the overall cardiac function of the study cohort was preserved (Table 4).

### 3.3. Atrial Fibrillation Status After Follow-Up

After a median follow-up period of 12 months, 16 cases (6.9%) of new-onset atrial fibrillation developed among the 232 baseline patients.

**Figure 3:**
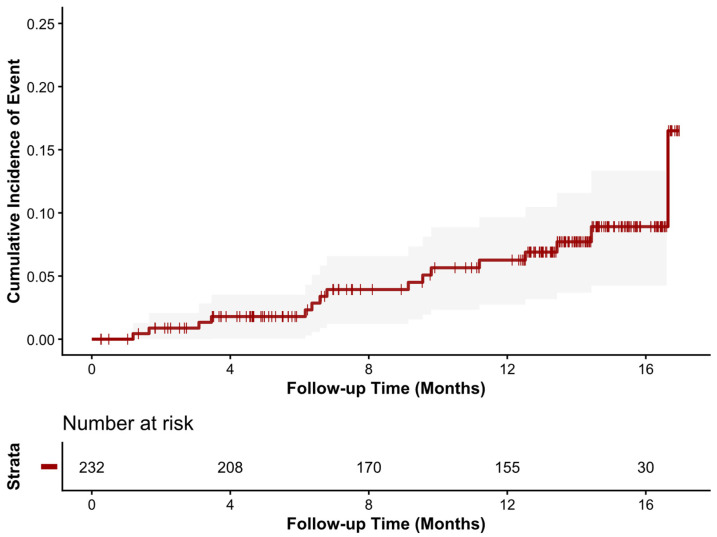
Kaplan–Meier curve showing cumulative probability of atrial fibrillation over time. The curve depicts a gradual increase in the cumulative incidence of AF, reaching nearly 10% after the median 12-month follow-up period. The shaded area represents the 95% confidence intervals, and the data table below tracks the declining number of patients at risk over the 17-month observation window.

The Kaplan–Meier curve demonstrated a gradual increase in the cumulative incidence of AF over time, reaching nearly 10% after 12 months. The 95% confidence intervals were narrow initially but widened progressively, indicating that the precision of the estimate decreased as the number of remaining patients diminished. The patient count at risk declined significantly over time: from 232 at baseline to 208 at 4 months, 155 at 12 months, 30 at 16 months, and finally just 19 at 17 months (Figure 3). To ensure full transparency regarding this attrition, the censoring mechanisms throughout the follow-up period were systematically accounted for. Out of the initial 232 patients, 16 (6.9%) reached the primary endpoint of new-onset AF. The median follow-up duration was 12 months (IQR: 6.75 to 13 months). Because a significant proportion of patients completed their clinical tracking within this interquartile range, the sharp drop in the number at risk beyond 12 months was largely expected. Among the remaining 216 censored patients, 176 were administratively censored. Furthermore, 6 died from other causes (competing risks) prior to developing AF, and 34 were strictly lost to follow-up. Therefore, the dramatic decrease in the number of patients at risk towards the later months was primarily an artifact of administrative censoring due to staggered recruitment, rather than a massive loss to follow-up.

### 3.4. Role of NT-proBNP and Predictors of Atrial Fibrillation

**Figure 4:**
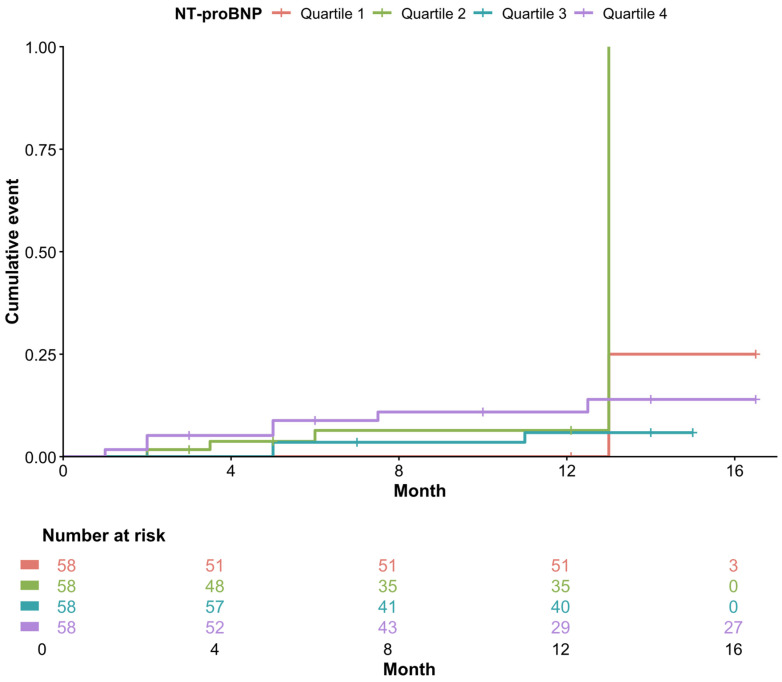
Comparison of Kaplan–Meier curves for new-onset atrial fibrillation cases by initial NT-proBNP quartile. The visual analysis and corresponding formal tests reveal no statistically significant difference (*p* = 0.3) or linear trend (*p* = 0.28) in the cumulative incidence of AF across the four biomarker quartiles, indicating that baseline NT-proBNP levels did not act as a predictor in this cohort.

Initial analysis showed that baseline NT-proBNP levels were slightly higher in the group that developed AF (median 36.0 pmol/L [304.9 pg/mL], IQR: 7.47–162 pmol/L [63.3–1372.1 pg/mL]) compared to the group that did not (median 34.7 pmol/L [293.9 pg/mL], IQR: 7.22–157 pmol/L [61.2–1329.8 pg/mL]). However, this difference was not statistically significant (*p* = 0.327) by the Wilcoxon rank sum test. An analysis of Kaplan–Meier curves across NT-proBNP quartiles also revealed no significant difference between the groups (*p* = 0.3). Furthermore, a formal log-rank test for trend across the quartiles was performed, demonstrating no statistically significant linear trend (*p*-value for trend = 0.28) (Figure 4).

The results of the parsimonious model revealed that three factors remained statistically associated with AF risk:

Left atrial (LA) size: (HR = 1.21, 95% CI: 1.11–1.32, *p* < 0.001). Each millimeter increase in LA size raises the risk of developing AF by 21%.

History of diabetes mellitus: (HR = 3.84, 95% CI: 1.35–10.92, *p* = 0.011).

History of stroke: (HR = 4.65, 95% CI: 1.12–19.34, *p* = 0.034).

**Figure 5:**
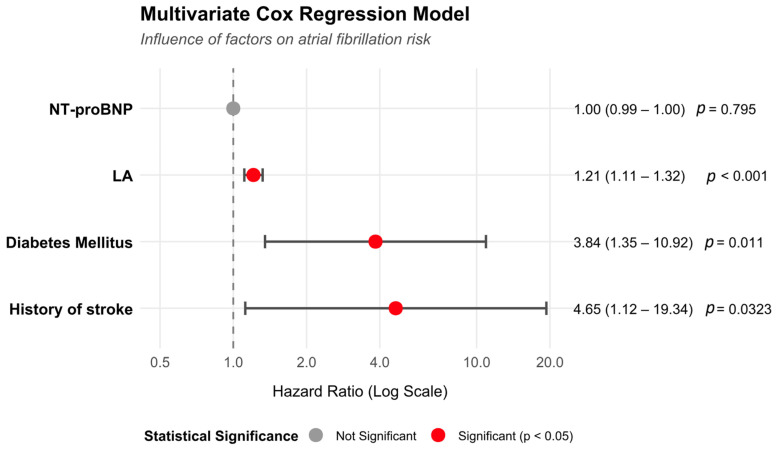
Forest plot of multivariate Cox regression analysis for factors associated with atrial fibrillation risk. The plot displays the hazard ratios and 95% confidence intervals for selected clinical predictors. Left atrial (LA) size, a history of diabetes mellitus, and a history of stroke were identified as statistically significant independent predictors of AF (*p* < 0.05), whereas baseline NT-proBNP concentration was not significant (*p* = 0.795). The dashed vertical line represents the line of no effect (Hazard Ratio = 1.0).

In contrast, baseline NT-proBNP levels remained statistically non-significant in predicting AF within this cohort (HR = 0.9997, 95% CI: 0.9972–1.0022, *p* = 0.795) (Table 5, Figure 5).

## 4. Discussion

This exploratory study was initially conducted with the hypothesis that high plasma NT-proBNP levels at hospital admission could serve as an independent predictor for the onset of AF in patients with non-AF arrhythmias, alongside traditional structural and clinical risk factors. The primary objective was to evaluate the role of NT-proBNP in predicting new-onset AF. After excluding cases with baseline AF and severe renal impairment, 232 non-AF patients were included in the analysis and followed for a median duration of 12 months.

Although NT-proBNP levels tended to be higher in the group that developed AF, the admission NT-proBNP concentration did not demonstrate a statistically significant, independent predictive role for new-onset AF in the multivariable Cox model (HR = 0.9997; *p* = 0.795). Instead, LA size, a history of diabetes mellitus, and a history of stroke emerged as suggestive, hypothesis-generating predictors for predicting new-onset AF. Specifically, a history of stroke increased the risk of AF by nearly 5 times (HR = 4.65), while diabetes increased it by nearly 4 times (HR = 3.84), and each millimeter increase in LA size raised the risk by 21% (HR = 1.21).

Our findings differ significantly from many previous studies that have demonstrated a strong predictive role of NT-proBNP for new-onset AF [1,6,16,17,18,19,20]. For instance, studies in the general population or in heart failure patients often found an independent association between NT-proBNP and AF risk [21,22]. This discrepancy can be explained by several factors.

First, our study population consisted of patients with pre-existing non-AF arrhythmias who were admitted with a diverse spectrum of symptoms, with syncope (59.9%) and dizziness (55.6%) being the most common, rather than typical palpitations. This may suggest a more complex underlying pathophysiology, where the baseline arrhythmias were already severe enough to cause hemodynamic disturbances, and NT-proBNP may be reflecting myocardial wall stress from these arrhythmias rather than directly predicting the conversion to AF.

Second, the median follow-up period of our study was 12 months, whereas some other studies with longer follow-up durations may have been better able to detect a clearer association.

Nevertheless, our results reinforce the role of left atrial (LA) size as a strong and consistent risk factor for AF, which has been widely established in the literature [5,23,24]. Similarly, the association between diabetes and AF risk is well-documented, through mechanisms such as systemic inflammation, myocardial fibrosis, and microvascular disease [25,26,27]. The finding of a history of stroke as a suggestive predictor of AF (HR = 4.65) is particularly noteworthy. This could suggest that patients with a prior stroke may have had undiagnosed paroxysmal AF or other severe underlying risk factors that make them more susceptible to AF.

To contextualize these findings, the lack of independent predictive utility for NT-proBNP in our multivariable model must be systematically interpreted through a multidimensional pathophysiological lens, contrasting our data with prior cohorts:

Biological Specificity and Pathophysiology: NT-proBNP is primarily synthesized and secreted by the ventricular myocardium in response to ventricular wall stress and volume overload. Recent meta-analyses, such as the 2025 study by Wang et al., confirm its strong association with AF in broad populations [4]; however, in a highly specific arrhythmia cohort, global ventricular stress may not directly correlate with localized atrial fibrillogenesis. Once a direct marker of atrial structural remodeling (LA size) is accounted for in the multivariable model, the non-specific ventricular signal of NT-proBNP loses its independent predictive power for an atrial-specific electrical outcome [10,28].

Cohort-Specific Factors: Unlike studies comprising primarily heart failure populations or general community cohorts [21,22], our cohort represents a unique electrophysiological subset. The study design systematically excluded end-stage renal disease (a major driver of NT-proBNP accumulation), and the prevalence of established heart failure was only 18.0%. More importantly, the profound heterogeneity of the baseline non-AF arrhythmias—ranging from severe AV blocks to sick sinus syndrome—introduces highly variable baseline hemodynamic profiles. This extreme heterogeneity acts as a powerful confounder that dilutes the prognostic specificity of NT-proBNP for AF.

Sampling Timing and Acute Hemodynamic Stress: Furthermore, blood samples in our study were drawn acutely upon hospital admission. In this acute symptomatic setting (characterized predominantly by syncope and dizziness), an elevated NT-proBNP level is highly likely to reflect the acute, transient hemodynamic stress and compensatory responses triggered by the primary presenting arrhythmia itself. Consequently, these acute levels do not reliably represent the steady-state, chronic atrial myopathy and continuous structural remodeling that traditionally predispose a patient to future new-onset AF.

Furthermore, the methodological handling of NT-proBNP warrants specific consideration. While log-transformation is a standard approach for highly skewed biomarkers to satisfy mathematical assumptions of linearity, forcing such data into normality in this specific cohort would only serve statistical compliance without yielding meaningful biomedical significance. The extreme heterogeneity of our data realistically reflects the clinical reality of a non-AF arrhythmia population. Importantly, this skewed distribution was robustly accounted for in our quartile-based Kaplan–Meier analysis (Figure 4), which yielded a completely non-significant overall log-rank test (*p* = 0.3) and no significant linear trend across strata. We acknowledge the methodological concern that categorizing a continuous biomarker into quartiles inherently risks diluting true predictive signals. However, it is crucial to emphasize that our primary multivariable Cox regression model strictly utilized NT-proBNP as a continuous variable to maximize data retention and prevent such dilution. The combined architectural consistency between the non-significant continuous Cox model and the quartile visualization reinforces that the lack of independent predictive value for NT-proBNP in this cohort is a true reflection of the clinical characteristics, rather than an artifact of suboptimal statistical modeling.

A low number of events and statistical power constraints: Only 16 new-onset AF cases were recorded out of 232 patients (an incidence rate of approximately 6.9%) after a median follow-up of 12 months. This observed rate is markedly lower than the 36.8% incidence derived from historical reference data used in our original sample size calculation. This strict event-to-variable ratio severely underpowers our multivariable Cox proportional hazards analysis, restricting the statistical capacity to identify an independent predictive signal for baseline NT-proBNP levels. Therefore, the lack of statistical significance for NT-proBNP in this cohort must be interpreted with caution, as it may reflect a lack of statistical power rather than the absolute absence of a biological association. Consequently, our findings should be treated as hypothesis-generating, and larger multi-center registries with a higher total event volume are required to clarify this relationship definitively.

Conversely, the prominence of left atrial size is physiologically sound. LA enlargement is a consequence of chronic elevated atrial filling pressure and a direct marker of atrial remodeling, which increases the risk of developing re-entry circuits and ectopic foci, leading to AF [29,30]. Similarly, diabetes induces diabetic cardiomyopathy, including atrial fibrosis and endothelial dysfunction, creating a favorable substrate for AF [25,31,32,33,34]. A history of stroke, with a high HR, may reflect a patient group with pre-existing cerebrovascular damage often accompanied by severe underlying cardiovascular conditions (including undiagnosed paroxysmal AF) or a high thrombotic risk.

These identified predictors align closely with established clinical frameworks for AF prediction and risk stratification. For instance, the HATCH score (Heart failure, Age, TIA/Stroke, COPD, Hypertension)—used to predict AF progression—and the CHA_2_DS_2_-VASc score both heavily weight a history of stroke/TIA and diabetes mellitus as critical clinical determinants of atrial arrhythmogenesis and thromboembolic risk. Furthermore, given the lack of independent predictive value for NT-proBNP in our multivariable model, it becomes evident that these clinical and echocardiographic factors do not merely offer incremental predictive value beyond biomarkers. Rather, they provide the primary, indispensable predictive value in this specific non-AF arrhythmia population, reinforcing the continued reliance on traditional structural and clinical risk paradigms over isolated biomarker screening.

The results of this study provide a nuanced understanding of AF predictors in a specific and clinically relevant patient population. While NT-proBNP was not an independent predictor in this cohort, the findings underscore that a careful evaluation of classical clinical factors and left atrial size remains central to AF risk stratification.

For patients with non-AF arrhythmias who are admitted to the hospital, particularly with symptoms like syncope and dizziness, meticulous screening for a history of diabetes, stroke, and an echocardiographic assessment of LA size are critically important. Patients with these risk factors should be monitored more aggressively for AF, which may include extended Holter ECG or more advanced cardiac rhythm monitoring devices. This has significant implications for personalizing risk management and stroke prevention strategies related to AF.

This research has several limitations that must be explicitly acknowledged as major threats to statistical inference. The most critical limitation is the small absolute number of outcome events, with only 16 new-onset AF cases recorded out of 232 patients. This low event rate drastically constrained the degrees of freedom available for multivariable prognostic modeling. Initial attempts to include a comprehensive array of baseline covariates led to severe overfitting and coefficient instability, generating wide and imprecise hazard estimates. Although we successfully mitigated this by adopting a restrictive, parsimonious prespecified model focusing on four key parameters, the lower-than-expected event rate materially restricts our statistical power. Consequently, our findings regarding independent clinical predictors must be interpreted with caution and treated as hypothesis-generating rather than definitive. Furthermore, while we recognize that different underlying arrhythmia substrates (e.g., sick sinus syndrome versus ventricular ectopy) inherently possess different risks for progressing to AF, the low event rate (*n* = 16) precluded us from adjusting for these specific baseline arrhythmia subtypes in our multivariable Cox model. The inability to control for the primary arrhythmia diagnosis means that some unmeasured confounding related to the specific electrophysiological substrate may remain, which should be addressed in larger, adequately powered multicenter cohorts. Additionally, our structural assessment was constrained by the available baseline data. While current international guidelines prefer the left atrial volume index (LAVI) over simple diameter as a more precise indicator of AF-related structural remodeling, our prospective protocol relied on the standard AP diameter uniformly recorded across all participants at hospital admission. The lack of LAVI data is an acknowledged limitation, and incorporating volume indexing remains an important area for improvement in future registry studies. Another limitation is the intermittent follow-up and a notable rate of loss to follow-up; the substantial decrease in the number of study participants over time (down to only 19 patients at 17 months) may introduce bias into the survival estimates and reduce the reliability of the results in the later stages of the study. Moreover, the presence of competing risks—specifically the 6 patients who died from other causes prior to developing AF—must be acknowledged. As we utilized standard Kaplan–Meier analysis rather than Cumulative Incidence Functions (CIF) or Fine-Gray subdistribution hazard models due to the extremely low event counts, our survival estimates may theoretically overestimate the cumulative incidence of AF. This competing risk bias represents a methodological limitation of our current analysis. Additionally, due to the severe constraints imposed by the low absolute number of primary events (*n* = 16), model discrimination metrics such as Harrell’s C-index were intentionally omitted. In small event-to-variable ratios, internal estimation of discrimination capacity is highly unstable and prone to severe optimism bias; thus, reporting these metrics without robust external validation could lead to clinical over-interpretation of this exploratory model. Furthermore, the detection of AF relied solely on ECG and Holter ECG, which may have missed short or asymptomatic episodes of paroxysmal AF. The use of more prolonged monitoring devices like implantable loop recorders could improve detection but was not feasible in the context of this study. Finally, this is a single-center study, which may limit the generalizability of the results to more diverse patient populations or other medical centers with different clinical characteristics and management protocols.

## 5. Conclusions

Our study concludes that baseline NT-proBNP is not an independent predictor for new-onset AF in patients with non-AF arrhythmias. However, traditional clinical factors play a highly significant role. Specifically, left atrial size, a history of diabetes mellitus, and a history of stroke were identified as strong, independent predictors. These findings underscore the necessity of a comprehensive assessment of classical risk factors for AF screening and management, particularly in high-risk patients.

## Figures and Tables

**Table 1:** General characteristics of 232 non-atrial fibrillation patients.

	Total (*n* = 232)		Male (*n* = 124)		Female (*n* = 108)	
	Mean ± SD	Median (IQR)	Mean ± SD	Median (IQR)	Mean ± SD	Median (IQR)
Age (years)	63.7 ± 14.0	67 (53–76)	60.4 ± 16.7	67 (53–76)	68.1 ± 13.9	70 (58–77)
Height (m)	1.59 ± 0.09	1.60 (1.55–1.65)	1.63 ± 0.05	1.60 (1.55–1.65)	1.55 ± 0.10	1.55 (1.50–1.56)
Weight (kg)	57.7 ± 10.5	58 (50–65)	61.4 ± 10.2	58 (50–65)	53.3 ± 8.93	53 (48–60)
BMI (kg/m^2^)	22.5 ± 3.34	22.4 (20.4–24.1)	22.8 ± 3.27	22.4 (20.4–24.1)	22.1 ± 3.37	22.1 (20.0–23.6)
Systolic BP (mmHg)	129 ± 19.6	130 (120–140)	128 ± 20.5	130 (120–140)	130 ± 18.7	130 (120–140)
Diastolic BP (mmHg)	75.1 ± 10.2	74.5 (70–80)	74.2 ± 10.9	74.5 (70–80)	75.9 ± 9.31	80 (70–80)

**Table 2:** Presenting symptoms at admission of non-atrial fibrillation arrhythmia patients (*n* = 232).

Symptom	Frequency (*n*)	Percentage (%)
Syncope	139	59.9
Dizziness	129	55.6
Dyspnea (Shortness of breath)	51	21.9
Palpitations	32	13.8
Fatigue	32	13.8
Chest pain	27	11.6
Chest tightness	10	4.31
Lightheadedness/Giddiness/Presyncope	10	4.31
Weakness	7	3.01
Other (difficulty swallowing, hand tremors, slow and difficult speech, abdominal pain, vomiting, hiccups, leg edema, breathlessness, numbness in limbs, pacemaker battery depletion)	12	5.17

**Table 3:** Distribution of primary baseline non-AF arrhythmia diagnoses (*n* = 232).

Arrhythmia Diagnosis	Frequency (*n*)	Percentage (%)
Atrioventricular (AV) block	88	37.9
Sick sinus syndrome	69	29.7
Ventricular arrhythmias (VT/VF)	22	9.5
Heart failure with intraventricular conduction delay (e.g., LBBB)	22	9.5
Brugada syndrome	17	7.3
Other	14	6.0
Total	232	100

**Table 4:** Selected laboratory and echocardiographic parameters of the study group.

*n*= 232	Median (IQR)
Laboratory tests	
HGB (g/L)	128 (116–138)
HCT (%)	38.5 (35.2–41.1)
WBC (G/L)	8.20 (6.75–10.2)
PLT (G/L)	192 (138–241)
INR	1.04 (1.01–1.11)
Creatinine (mg/dL)	0.87 (0.75–1.03)
eGFR (mL/min/1.73 m^2^)	83.9 (64.3–96.7)
Free T4 (pg/mL)	12.3 (11.0–13.4)
TSH (mIU/L)	1.41 (0.80–2.23)
Cholesterol (mg/dL)	163 (133–191)
HDL-Cholesterol (mg/dL)	41 (35–49)
LDL-Cholesterol (mg/dL)	96 (72–124)
Triglyceride (mg/dL)	136 (94–187)
Echocardiography	
LA (mm)	31 (28–35)
EF (%)	65 (57–71)
EDV (mL)	102 (85–129)
ESV (mL)	36 (28–51)
LVEDD (mm)	47 (44–52)
LVESD (mm)	30 (27–34)

**Table 5:** Multivariate Cox regression model assessing the influence of factors on atrial fibrillation risk.

Variable	HR (95% CI)	*p*-Value
NT-proBNP	1.00 (0.99–1.00)	0.795
LA	1.21 (1.11–1.32)	<0.001
Diabetes Mellitus	3.84 (1.35–10.92)	0.011
History of stroke	4.65 (1.12–19.34)	0.0323

## Data Availability

The datasets generated and/or analyzed during the current study are not publicly available due to policies protecting participant privacy and confidentiality, but are available from the corresponding author (Linh Ha Khanh Duong, khanhlinh175@gmail.com) on request.

## Abbreviations

The following abbreviations are used in this manuscript:

AF	Atrial Fibrillation
BMI	Body Mass Index
ECG	Electrocardiogram
EF	Ejection Fraction
eGFR	estimated Glomerular Filtration Rate
HR	Hazard Ratio
IQR	Interquartile Range
LA	Left Atrial
NT-proBNP	N-terminal pro-B-type natriuretic peptide

## References

[1] Schnabel R.B., Yin X., Gona P., Larson M.G., Beiser A.S., McManus D.D., Newton-Cheh C., Lubitz S.A., Magnani J.W., Ellinor P.T. (2015). 50 year trends in atrial fibrillation prevalence, incidence, risk factors, and mortality in the Framingham Heart Study: A cohort study. Lancet.

[2] Kornej J., Borschel C.S., Benjamin E.J., Schnabel R.B. (2020). Epidemiology of Atrial Fibrillation in the 21st Century: Novel Methods and New Insights. Circ. Res..

[3] January C.T., Wann L.S., Alpert J.S., Calkins H., Cigarroa J.E., Cleveland J.C., Conti J.B., Ellinor P.T., Ezekowitz M.D., Field M.E. (2014). 2014 AHA/ACC/HRS guideline for the management of patients with atrial fibrillation: A report of the American College of Cardiology/American Heart Association Task Force on Practice Guidelines and the Heart Rhythm Society. J. Am. Coll. Cardiol..

[4] Wang W., Zhou T., Li J., Yuan C., Li C., Chen S., Shen C., Gu D., Lu X., Liu F. (2025). Association between NT-proBNP levels and risk of atrial fibrillation: A systematic review and meta-analysis of cohort studies. Heart.

[5] Bakir E.O., Yurdam F.S., Dolu A.K., Aguloglu S. (2025). The relationship between the left atrium/left ventricle ratio and atrial fibrillation in patients with ischemic stroke without significant left atrial enlargement. Int. J. Cardiovasc. Imaging.

[6] Lancini D., Sun J., Mylonas G., Boots R., Atherton J., Prasad S., Martin P. (2024). Predictors of New Onset Atrial Fibrillation Burden in the Critically Ill. Cardiology.

[7] O’Neal W.T., Efird J.T., Yeboah J., Nazarian S., Alonso A., Heckbert S.R., Soliman E.Z. (2014). Brachial flow-mediated dilation and incident atrial fibrillation: The multi-ethnic study of atherosclerosis. Arter. Arterioscler. Thromb. Vasc. Biol..

[8] Galea R., Cardillo M.T., Caroli A., Marini M.G., Sonnino C., Narducci M.L., Biasucci L.M. (2014). Inflammation and C-reactive protein in atrial fibrillation: Cause or effect?. Tex. Heart Inst. J..

[9] Sandefur C.C., Jialal I. Atrial Natriuretic Peptide. National Library of Medicine, National Center for Biotechnology Information 2023. https://www.ncbi.nlm.nih.gov/books/NBK562257/.

[10] Novack M.L., Zubair M. Natriuretic Peptide B Type Test. National Library of Medicine, National Center for Biotechnology Information 2023. https://www.ncbi.nlm.nih.gov/books/NBK556136/.

[11] Nasab Mehrabi E., Toupchi-Khosroshahi V., Athari S.S. (2023). Relationship of atrial fibrillation and N terminal pro brain natriuretic peptide in heart failure patients. ESC. Heart Fail..

[12] Girerd N., Levy D., Duarte K., Ferreira J.P., Ballantyne C., Collier T., Pizard A., Bjorkman J., Butler J., Clark A. (2023). Protein Biomarkers of New-Onset Heart Failure: Insights From the Heart Omics and Ageing Cohort, the Atherosclerosis Risk in Communities Study, and the Framingham Heart Study. Circ. Heart Fail..

[13] Staerk L., Preis S.R., Lin H., Lubitz S.A., Ellinor P.T., Levy D., Benjamin E.J., Trinquart L. (2020). Protein Biomarkers and Risk of Atrial Fibrillation: The FHS. Circ. Arrhythm. Electrophysiol..

[14] Lindberg T., Wimo A., Elmstahl S., Qiu C., Bohman D.M., Sanmartin Berglund J. (2019). Prevalence and Incidence of Atrial Fibrillation and Other Arrhythmias in the General Older Population: Findings From the Swedish National Study on Aging and Care. Gerontol. Geriatr. Med..

[15] Kotalczyk A., Lip G.Y., Calkins H. (2021). The 2020 ESC Guidelines on the Diagnosis and Management of Atrial Fibrillation. Arrhythm. Electrophysiol. Rev..

[16] Ardhianto P., Yuniadi Y. (2019). Biomarkers of Atrial Fibrillation: Which One Is a True Marker?. Cardiol. Res. Pr. Pract..

[17] Borschel C.S., Ohlrogge A.H., Geelhoed B., Niiranen T., Havulinna A.S., Palosaari T., Jousilahti P., Rienstra M., van der Harst P., Blankenberg S. (2021). Risk prediction of atrial fibrillation in the community combining biomarkers and genetics. Europace.

[18] Manjer J., Carlsson S., Elmstahl S., Gullberg B., Janzon L., Lindstrom M., Mattisson I., Berglund G. (2001). The Malmo Diet and Cancer Study: Representativity, cancer incidence and mortality in participants and non-participants. Eur. J. Cancer Prev..

[19] Patton K.K., Ellinor P.T., Heckbert S.R., Christenson R.H., DeFilippi C., Gottdiener J.S., Kronmal R.A. (2009). N-terminal pro-B-type natriuretic peptide is a major predictor of the development of atrial fibrillation: The Cardiovascular Health Study. Circulation.

[20] Patton K.K., Heckbert S.R., Alonso A., Bahrami H., Lima J.A., Burke G., Kronmal R.A. (2013). N-terminal pro-B-type natriuretic peptide as a predictor of incident atrial fibrillation in the Multi-Ethnic Study of Atherosclerosis: The effects of age, sex and ethnicity. Heart.

[21] Schnabel R.B., Larson M.G., Yamamoto J.F., Sullivan L.M., Pencina M.J., Meigs J.B., Tofler G.H., Selhub J., Jacques P.F., Wolf P.A. (2010). Relations of biomarkers of distinct pathophysiological pathways and atrial fibrillation incidence in the community. Circulation.

[22] Li L., Selvin E., Lutsey P.L., Hoogeveen R.C., O’Neal W.T., Soliman E.Z., Chen L.Y., Alonso A. (2018). Association of N-terminal pro B-type natriuretic peptide (NT-proBNP) change with the risk of atrial fibrillation in the ARIC cohort. Am. Heart J..

[23] Suryabanshi A., Timilsina B., Shakya S., Khanal S., Yadav V., Joshi A. (2024). Left Atrial Enlargement as a Predictor of Atrial Fibrillation in Rheumatic Mitral Valve Disease: An Echocardiography-based Retrospective Cross-sectional Study. J. Nepal. Health Res. Counc..

[24] Saadeh R., Abu Jaber B., Alzuqaili T., Ghura S., Al-Ajlouny T., Saadeh A.M. (2024). The relationship of atrial fibrillation with left atrial size in patients with essential hypertension. Sci. Rep..

[25] Ugowe F.E., Jackson L.R., Thomas K.L. (2019). Atrial Fibrillation and Diabetes Mellitus: Can We Modify Stroke Risk Through Glycemic Control?. Circ. Arrhythm. Electrophysiol..

[26] Fatemi O., Yuriditsky E., Tsioufis C., Tsachris D., Morgan T., Basile J., Bigger T., Cushman W., Goff D., Soliman E.Z. (2014). Impact of intensive glycemic control on the incidence of atrial fibrillation and associated cardiovascular outcomes in patients with type 2 diabetes mellitus (from the Action to Control Cardiovascular Risk in Diabetes Study). Am. J. Cardiol..

[27] Dublin S., Glazer N.L., Smith N.L., Psaty B.M., Lumley T., Wiggins K.L., Page R.L., Heckbert S.R. (2010). Diabetes mellitus, glycemic control, and risk of atrial fibrillation. J. Gen. Intern. Med..

[28] Cao Z., Jia Y., Zhu B. (2019). BNP and NT-proBNP as Diagnostic Biomarkers for Cardiac Dysfunction in Both Clinical and Forensic Medicine. Int. J. Mol. Sci..

[29] Seckin O., Unlu S., Yalcin M.R. (2025). The hidden role of left atrial strain: Insights into end-organ damage in dipper and nondipper hypertension. J. Hum. Hypertens..

[30] Parajuli P., Alahmadi M.H., Ahmed A.A. Left Atrial Enlargement. National Library of Medicine, National Center for Biotechnology Information 2025. https://www.ncbi.nlm.nih.gov/books/NBK553096/.

[31] Leopoulou M., Theofilis P., Kordalis A., Papageorgiou N., Sagris M., Oikonomou E., Tousoulis D. (2023). Diabetes mellitus and atrial fibrillation-from pathophysiology to treatment. World J. Diabetes.

[32] Seyed Ahmadi S., Svensson A.M., Pivodic A., Rosengren A., Lind M. (2020). Risk of atrial fibrillation in persons with type 2 diabetes and the excess risk in relation to glycaemic control and renal function: A Swedish cohort study. Cardiovasc. Diabetol..

[33] Wang A., Green J.B., Halperin J.L., Piccini J.P. (2019). Atrial Fibrillation and Diabetes Mellitus: JACC Review Topic of the Week. J. Am. Coll. Cardiol..

[34] Aune D., Feng T., Schlesinger S., Janszky I., Norat T., Riboli E. (2018). Diabetes mellitus, blood glucose and the risk of atrial fibrillation: A systematic review and meta-analysis of cohort studies. J. Diabetes Complicat..

